# ﻿A first revision of the *Andrena* of Iraq (Hymenoptera, Andrenidae), with the description of two new species from Iraqi Kurdistan and additional records from surrounding countries

**DOI:** 10.3897/zookeys.1205.120033

**Published:** 2024-07-01

**Authors:** Thomas J. Wood, Halgurd R. Ismael, Daniele Baiocchi, Mudhafar I. Hamad, Tahseen T. Bapir, Marco Selis

**Affiliations:** 1 Naturalis Biodiversity Center, 2333 CR, Leiden, Netherlands Naturalis Biodiversity Center Leiden Netherlands; 2 Department of Plant Protection, College of Agricultural Engineering Sciences, University of Duhok, Duhok, Kurdistan Region, Iraq University of Duhok Duhok Iraq; 3 Via Matteo Babini 26, 00139, Roma, Italy Unaffiliated Roma Italy; 4 Khabat Technical Institute, Erbil Polytechnic University, Erbil, Kurdistan Region, Iraq Erbil Polytechnic University Erbil Iraq; 5 Via dei Tarquini 22, 01100 VT, Viterbo, Italy Unaffiliated Viterbo Italy

**Keywords:** Middle East, pan trap, solitary bees, taxonomy, understudied fauna

## Abstract

Iraq is a large country in the Middle East region that borders both Turkey and Iran, countries known to host two of the largest bee faunas globally, as expected for a group of insects that favour dry to Mediterranean climates. Despite this huge regional species richness, the bee fauna of Iraq is chronically understudied and poorly known, both in relative and absolute terms. This is true for the hyper-speciose bee genus *Andrena*, for which only 17 species have been previously published for Iraq. This work is the first modern contribution to the revision of the *Andrena* fauna of Iraq. Based on new specimen collections in Duhok Governorate (Iraqi Kurdistan) during 2023, a revised total of 59 *Andrena* species for Iraq (42 species recorded for the first time) is presented, including the description of two new species: Andrena (Aciandrena) duhokensis Wood, **sp. nov.** and Andrena (Notandrena) baiocchii Wood, **sp. nov.** The unknown males of A. (Micrandrena) elam Wood, 2022, A. (Micrandrena) obsidiana Wood, 2022, and A. (Notandrena) ayna Wood, 2023 are described. *Andrenabakrajoensis* Amin & Mawlood, 2019, **syn. nov.** is synonymised with A. (Holandrena) variabilis Smith, 1853. Additional records are presented from nearby Middle Eastern countries, particularly Lebanon. These results highlight the fundamentally understudied nature of the Iraqi *Andrena* fauna.

## ﻿Introduction

*Andrena* is the second largest genus of bees with approximately 1,700 species following recent revisions (e.g., Pisanty et al. 2022a; Wood and Monfared 2022; Wood 2023, 2024). *Andrena* are known for their explosive radiation and rapid rate of speciation (Bossert et al. 2022), and are likely to have evolved in the Middle East region ca 25 million years ago (Pisanty et al. 2022b). In line with this expected point of origin, the *Andrena* faunas of several Middle Eastern countries are very large, with ca 220 species known from Israel, 215 species known from Iran, and 388 species known from Turkey (Pisanty et al. 2022a; Wood and Monfared 2022; Wood 2023, 2024; Wood, unpublished data). The *Andrena* fauna of Syria is also large, but has not been comprehensively revised, and many species are present which have not yet had their occurrence formally published, with ~ 154–166 species known but not yet fully demonstrated or confirmed (Wood 2020; Wood et al. 2020; Pisanty et al. 2022a; Wood and Monfared 2022; Wood, unpublished data).

In contrast, the published *Andrena* fauna of the country of Iraq is extremely small, with only 17 species mentioned across various literature sources (Morice 1921a, 1921b; Derwesh 1965; Warncke 1969; Khalaf and Al-Omar 1974; Moalif 1994; Gusenleitner and Schwarz 2002; Scheuchl and Willner 2016; Augul 2018; Amin and Mawlood 2019; Ascher and Pickering 2023), some of which primarily cite previous publications without adding any new information, or which never present detailed specimen records at all. The Iraqi *Andrena* fauna has very rarely received dedicated attention, most frequently with species listed without supporting specimens or mentioned museum depositories, in strong contrast to neighbouring Turkey (e.g., Warncke 1965, 1974, 1975; Scheuchl and Gusenleitner 2009; Scheuchl and Hazir 2012; Hazir et al. 2014; Wood 2023) and to a lesser extent Iran and Syria (e.g., Popov 1967; Khodarahmi Ghahnavieh and Monfared 2019; Wood et al. 2020; Wood 2020; Radchenko et al. 2021; Wood and Monfared 2022).

Given the very high *Andrena* species richness found in nearby countries, it is clearly implausible that the Iraqi *Andrena* fauna is so small. In order to counter this knowledge deficit, we conducted new collections in northern Iraq during spring 2023, and critically reviewed the literature and museum collections in order to produce an updated faunal total.

## ﻿Materials and methods

**Figure 1.**
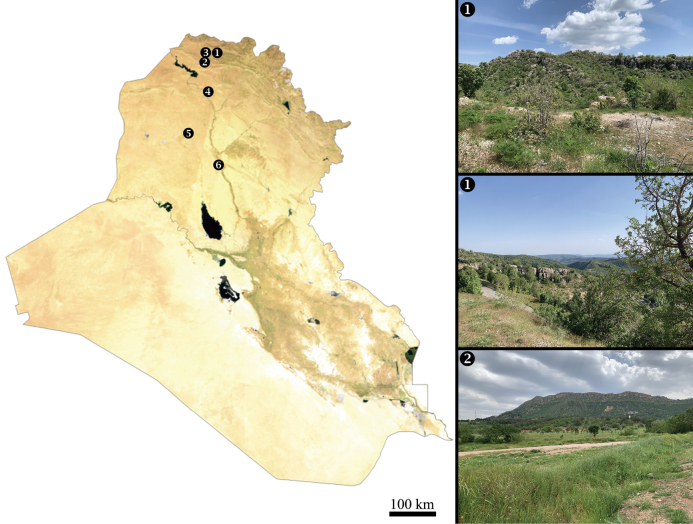
Map of Iraq with novel data sites marked. New data locations: 1. Mount Gara, south of Sarsing, Duhok Governorate; 2. Besereh, Bablo, Duhok Governorate; 3. Mangesh, Duhok Governorate; 4. Mosul, Nineveh Governorate; 5. Hatra, Nineveh Governorate; 6. Baiji, Saladin Governorate. Landscape photographs correspond to localities 1 and 2.

**Figure 2.**
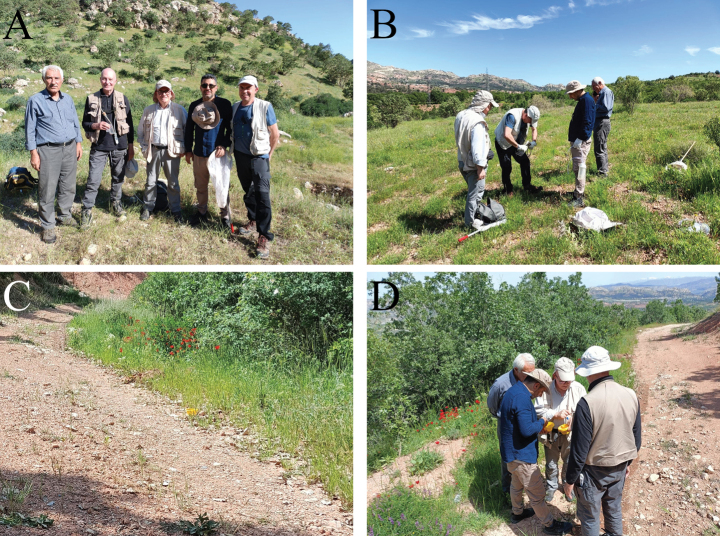
Sampling participants in Duhok Governorate during May 2023 **A** sampling participants from left to right: Mudhafar Hamad, Daniele Baiocchi, Gianluca Magnani, Halgurd Ismael, Pierpaolo Rapuzzi **B** selection of sampling sites **C** yellow pan trap placed adjacent to vegetation **D** pan trap collection and inspection.

*Andrena* specimens were collected from various localities in Duhok province, Iraq (Kurdistan region) during May 2023 (Fig. 1: locations 1, 2, and 3). Specimens were collected using yellow pan traps which were filled with water and placed adjacent to vegetation (Fig. 2C).

Morphological terminology follows Michener (2007). The following abbreviations are used in the species descriptions: **A** = antennal segments, **S** = metasomal sterna, and **T** = metasomal terga. In diagnoses, the defining characters of a species are given, with those of the indicated comparison species given in parentheses. Subgeneric concepts follow Pisanty et al. (2022b). Specimens were measured from the centre of the clypeus at the front of the head to the apical tip of the metasoma to the nearest 0.5 mm. In the faunal list, taxa are presented alphabetically, first by subgenus and then by species. In the full faunal list, species entries followed by an asterisk (*) indicate the first record for Iraq. Likewise, for the global distributions of species, countries marked with an asterisk (*) indicate the first published record for that country. All specimens were identified by T.J. Wood.

Photographs were taken using an Olympus E-M1 Mark II with a 60 mm macro lens. Additional close-ups were taken with the addition of a Mitutoyo M Plan Apo 5X infinity corrected objective lens. Photographs were stacked using Helicon Focus B (HeliconSoft, Ukraine) and plates were prepared in GNU Image Manipulation Program (GIMP) 2.10. Post-processing of some images was made in Photoshop Elements (Adobe Systems, USA) in order to improve lighting to highlight specific characters. The map of Iraq was taken from GISGeography (2024).

### ﻿Abbreviations of depositories

**DUMAI** University of Duhok, Museum of Agriculture College, Duhok, Iraq

**MSVI** Personal collection of Marco Selis, Viterbo, Italy

**NHMUK** Natural History Museum, London, United Kingdom

**OÖLM** Oberösterreiches Landesmusum, Linz, Austria

**RMNH** Naturalis Biodiversity Center, Leiden, the Netherlands

**TJWC** Personal collection of Thomas J. Wood, Leiden, the Netherlands

## ﻿Results

### ﻿Full Iraqi faunal list

#### ﻿1. Andrena (Aenandrena) aeneiventris

Taxon classificationAnimaliaHymenopteraAndrenidae

Morawitz, 1872

05DE10E3-3402-5AA1-B648-5AA032B1991E

##### Literature records.

Gusenleitner and Schwarz (2002: dot map 11); Scheuchl and Willner (2016); Ascher and Pickering (2023).

##### Remarks.

We have not examined any specimens of this species, but the presence of this species in Iraq is highly plausible based on its known global distribution, the map records indicated by Gusenleitner and Schwarz (2002), and the presence of this species in neighbouring Turkey and Iran.

##### Distribution.

West and Central Palearctic (Gusenleitner and Schwarz 2002; Osytshnjuk et al. 2005).

#### ﻿2. Andrena (Notandrena) aerinifrons

Taxon classificationAnimaliaHymenopteraAndrenidae

Dours, 1873 *

1F1263ED-5BE2-5DE7-9884-0B0763AAE564

##### Material examined.

**Iraq**: Mosul, edges of a river, 7.iv.1988, 1♂, leg. Olejníček, OÖLM; **Lebanon**: Balbek-Hermel, Sefri, Haouch Snaid, AUB farm, 33.9244°N, 36.0754°E, 1000 m, 6.iv.2023, 1♂, 8♀, leg. T.J. Wood, TJWC.

##### Remarks.

The status of the subspecies levantina Hedicke, 1938 (which nominally occurs in the Middle East) is unclear, and may not be merited, although *A.aerinifrons* sensu lato shows high and difficult-to-interpret intraspecific variation in its mitochondrial DNA barcode (Wood, unpublished data).

##### Distribution.

*Andrenaaerinifrons* sensu lato is distributed from Iberia and North Africa to the Middle East including Iraq* and Iran (Gusenleitner and Schwarz 2002; Wood and Monfared 2022).

#### ﻿3. Andrena (Taeniandrena) afzeliella

Taxon classificationAnimaliaHymenopteraAndrenidae

(Kirby, 1802)

C4757D8A-A314-573D-B873-99C2243E3715

##### Literature records.

Gusenleitner and Schwarz (2002: dot map 345, as *A.ovatula* (Kirby, 1802)); Ascher and Pickering (2023).

##### Remarks.

We have not examined any specimens of this historically confused species (see Praz et al. 2022), but the presence of this species in Iraq is highly plausible based on its known global distribution, the map records indicated by Gusenleitner and Schwarz (2002), and the presence of this species in neighbouring Turkey and Iran where it is abundant.

##### Distribution.

Somewhat unclear due to historical taxonomic confusion, but probably West and Central Palearctic (Praz et al. 2022; Wood and Monfared 2022; Ascher and Pickering 2023).

#### ﻿4. Andrena (Melandrena) albifacies

Taxon classificationAnimaliaHymenopteraAndrenidae

Alfken, 1927

450828FE-A19B-54E9-B66F-06101C9FCF51

##### Literature records.

Warncke (1969); Gusenleitner and Schwarz (2002: dot map 17); Grace (2010); Augul (2018); Wood and Monfared (2022); Ascher and Pickering (2023).

##### Remarks.

We have not examined any specimens of this species, but the presence of this species in Iraq is highly plausible based on its known global distribution, the mention of this species from southern Iraq (Warncke 1969), the map records indicated by Gusenleitner and Schwarz (2002), and the presence of this species in neighbouring Iran.

##### Distribution.

Morocco, Algeria, Tunisia, Libya, Egypt, Israel and West Bank, Jordan, Syria, Iraq, Iran (Warncke 1969; Gusenleitner and Schwarz 2002; Wood and Monfared 2022; Ascher and Pickering 2023).

#### ﻿5. Andrena (Truncandrena) albopicta

Taxon classificationAnimaliaHymenopteraAndrenidae

Radoszkowski, 1874

BB602A5E-8EA9-5501-B06E-2F68674FA196

##### Literature records.

Gusenleitner and Schwarz (2002: dot map 18); Grace (2010); Augul (2018); Ascher and Pickering (2023).

##### Remarks.

We have not examined any specimens of this species, but the presence of this species in Iraq is plausible: the species is present in south-eastern Turkey (see specimen records presented by Wood and Monfared 2022), and the dot map presented by Gusenleitner and Schwarz (2002) seems to indicate the presence of this species in northern Iraq. It is therefore tentatively accepted as present.

##### Distribution.

Turkey, Armenia, Iraq, Iran (Gusenleitner and Schwarz 2002; Ascher and Pickering 2023), note that previous records from European Russia were erroneous, see Proshchalykin et al. (2023).

#### ﻿6. Andrena (Melandrena) albopunctata

Taxon classificationAnimaliaHymenopteraAndrenidae

(Rossi, 1792)

3A76A028-B8AE-52BA-8008-A5CDD83F533E

##### Literature records.

Gusenleitner and Schwarz (2002: dot map 19); Ascher and Pickering (2023).

##### Remarks.

We have not examined any specimens of this species, but the presence of this species in Iraq is highly plausible based on its known global distribution, the map records indicated by Gusenleitner and Schwarz (2002), and the presence of this species in neighbouring Turkey and Iran.

##### Distribution.

West and Central Palearctic (Gusenleitner and Schwarz 2002; Osytshnjuk et al. 2008).

#### ﻿7. Andrena (incertae sedis) antilibanotica

Taxon classificationAnimaliaHymenopteraAndrenidae

Wood, 2020 *

B9AB82FB-A3D3-54FB-9200-536950677F10

##### Material examined.

**Iraq**: Duhok, Mt. Gara [S of Sarsing], 37.0158°N, 43.3506°E, 1912 m, 11.v.2023, 3♂, leg. D. Baiocchi, MSVI.

##### Distribution.

Syria, Turkey, Iraq*, Iran (Wood and Monfared 2022; Wood 2023).

#### ﻿8. Andrena (Chlorandrena) astica

Taxon classificationAnimaliaHymenopteraAndrenidae

Warncke, 1967 *

72B059BD-D1B7-58DD-AFC2-341D4FBD1BBF

##### Material examined.

**Iraq**: Duhok, Mt. Gara [S of Sarsing], 37.0158°N, 43.3506°E, 1912 m, 11.v.2023, 3♂, leg. D. Baiocchi, MSVI/TJWC; **Lebanon**: Bekaa, Qob Elias valley, 33.7989°N, 35.8192°E, 900 m, 5.iv.2023, 1♂, leg. M. Boustani, TJWC.

##### Distribution.

Greece, Turkey, Cyprus, Israel and West Bank, Lebanon*, Georgia, Iraq*, Iran (Schwenninger 2015; Wood and Monfared 2022).

#### ﻿9. Andrena (Notandrena) ayna

Taxon classificationAnimaliaHymenopteraAndrenidae

Wood, 2023 *

ADE0B8BD-8CFB-5334-8133-D34A7590CD32

##### Material examined.

**Iraq**: Duhok, Mt. Gara [S of Sarsing], 37.0158°N, 43.3506°E, 1912 m, 11.v.2023, 3♂, leg. D. Baiocchi, MSVI/TJWC.

##### Remarks and diagnosis.

**Figure 3.**
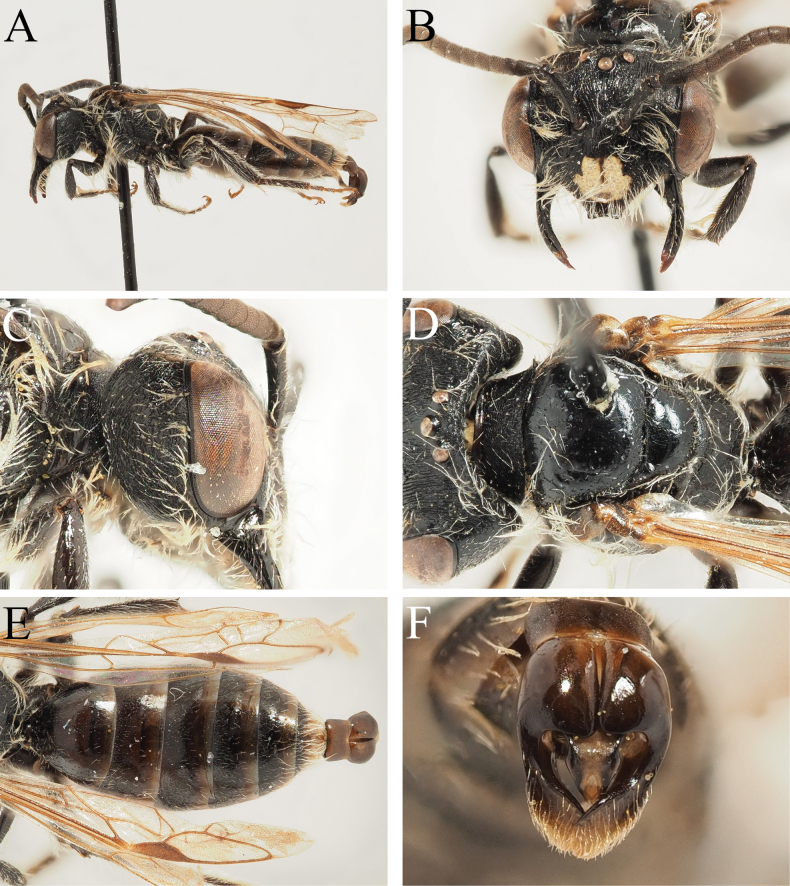
Andrena (Notandrena) ayna Wood, 2023 male **A** habitus, lateral view **B** face, frontal view **C** head, lateral view **D** scutum, dorsal view **E** terga, dorsal view **F** genital capsule, dorsal view.

Wood (2023) described *A.ayna* from south-eastern Turkey (province of Hakkâri) based on two female specimens. Three male specimens from northern Iraq are now available. They are recognisable as *Notandrena* due to the short and broad head (Fig. 3B; 1.25× wider than long), the apex of the clypeus narrowed and slightly upturned, the at least partially yellow-marked clypeus, the broadened and weakly carinate gena (Fig. 3C; broader than the diameter of the compound eye), and the pronotum with a strong humeral angle. As in the female sex, *A.ayna* is immediately recognisable due to the sculpture of the scutum and scutellum which are almost entirely smooth and shiny over their entirely area, with scattered and sparse punctures (Fig. 3D).

##### Description.

**Male. *Body length***: 6.5–7 mm (Fig. 3A). ***Head***: Dark, 1.25× wider than long (Fig. 3B). Clypeus variably yellow-marked, from medial ½ with yellow spot which does not extend to lateral margins to almost entirely dark with small pale dot medio-apically. Clypeus broadly flattened medially, surface densely and shallowly punctate, punctures separated by 0.5–1 puncture diameter, surface dull; apical margin narrow, projecting, anterior margin slightly upturned, weakly emarginate. Process of labrum tiny, trapezoidal, slightly wider than long, surface polished and shining. Gena 1.5× wider than diameter of compound eye, posterior margin weakly carinate, surface covered with curved striations (Fig. 3C); ocelloccipital distance subequal to diameter of lateral ocellus. Head covered with sparse light brownish pubescence, hairs not equalling length of scape. Antennae basally dark, A4–13 ventrally lightened by presence of greyish-brown scales; A3 exceeding A4, shorter than A4+5.

***Mesosoma***: Scutum and scutellum polished and shining over almost entire surface, scutum weakly shagreened anteriorly; scutum with scattered irregular punctures, punctures separated by 1–5 puncture diameters, scutellum almost impunctate (Fig. 3D). Pronotum with strongly produced humeral angle, deep vertical furrow with surface shining, remaining lateral face of propodeum with longitudinal striations. Mesepisternum irregularly microreticulate, weakly shining. Dorsolateral parts of propodeum shagreened, weakly shining, sculpture overlain by network of irregular raised rugosity; propodeal triangle laterally defined by fine straight carinae, internal surface with network of rugae radiating from base, propodeal triangle not strongly differentiated from remaining propodeum. Mesosoma covered with sparse light brown hairs, none equalling length of scape. Legs dark, apical tarsal segments paler orange-brown, pubescence whitish. Hind tarsal claws with inner tooth. Wings hyaline, stigma and venation orange, nervulus antefurcal.

***Metasoma***: Tergal discs dark, marginal areas broadly lightened hyaline brown-white (Fig. 3E). Terga with sculpture variable, disc of T1 smooth and shining, T2–4 with base weakly shagreened, sculpture disappearing medially, tergal margins without sculpture. T1 with disc sparsely punctate, punctures separated by 2–3 puncture diameters, discs of T2–4 with punctures separated by 1–2 puncture diameters, marginal areas impunctate. Tergal discs with scattered pale hairs, not forming hairbands. T6–7 with light brown hairs overlying pseudopygidial plate. S8 columnar, short, apical margin truncate, ventral surface with sparse short brown hairs. Genital capsule compact, gonocoxae with inner margins almost forming rounded 90° angle, very weakly projecting (Fig. 3F). Gonostyli narrow basally, strongly broadening and flattened apically, inner margin raised, outer surface with short golden-brown hairs. Penis valves occupying ½ space between gonostyli, outer margins slightly thickened, progressively narrowing apically.

##### Distribution.

South-eastern Turkey (Hakkâri) and northern Iraq* (Wood 2023).

#### ﻿10. Andrena (Notandrena) baiocchii

Taxon classificationAnimaliaHymenopteraAndrenidae

Wood, sp. nov. *

C0E8E128-7640-5F25-84F4-7A6A503D1AEC

https://zoobank.org/6005A148-DD6C-45CE-8284-4DB733F080F1

##### Material examined.

***Holotype*: Iraq**: Duhok, Mt. Gara [S of Sarsing], 37.0158°N, 43.3506°E, 1912 m, 11.v.2023, 1♀, leg. D. Baiocchi, RMNH. ***Paratypes*: Iraq**: Same information as holotype, 5♀, MSVI/RMNH/TJWC/DUMAI.

##### Diagnosis.

**Figure 4.**
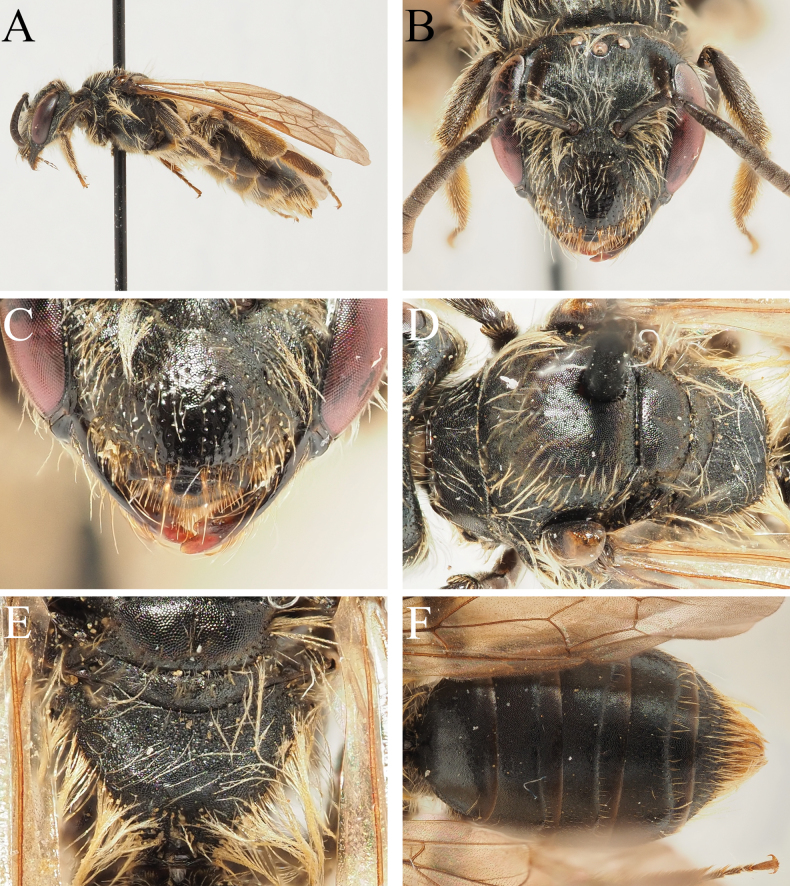
Andrena (Notandrena) baiocchii sp. nov. female **A** habitus, lateral view **B** face, frontal view **C** clypeus, frontal view detail **D** scutum, dorsal view **E** propodeum, dorsal view **F** terga, dorsal view.

*Andrenabaiocchii* can be recognised as part of the subgenus Notandrena Pérez, 1890 due to the dorsolateral angle of the pronotum with a strong transverse ridge (= pronotum with a strong humeral angle), dull impunctate terga (Fig. 4F; hence placing it closer to members of the former subgenus Carandrena Warncke, 1968), scutum with distinct but weakly shining to dull green-purple metallic reflections (Fig. 4D), hind tibiae which weakly but distinctly broaden medially and apically, resembling a crude isosceles triangle and covered with simple scopal hairs, and lack of any other distinctive characters. This combination of characters places it close to *A.schlettereri* Friese, 1896 (Central Europe to Turkey), *A.purpureomicans* Alfken, 1935 (Turkey), *A.trimarginata* (Radoszkowski, 1886) (= *A.zostera* Warncke, 1975; Middle East and Central Asia, see below), and *A.aerinifrons* Dours, 1873 sensu lato (Iberia, North Africa, and the Middle East). *Andreabaiocchii* can be separated from all these comparison species by the shape of the head which is almost round, only 1.1× wider than long (Fig. 4B; in comparison species is the head shorter, 1.2–1.4× wider than long) and the clypeus has contrasting surface sculpture, shagreened and weakly shining basally but becoming smooth and shining in its apical half AND with punctures becoming sparse, in the apical 1/3 of the clypeus with punctures separated by 1–3 puncture diameters (Fig. 4C). In comparison species the clypeus usually has a uniform sculpture, typically dull, never smooth and shining in its apical half (with the exception of *A.trimarginata*) and the clypeal punctures are consistently denser. *Andrenatrimarginata* can be separated by the overall shape of the head (clearly wider than long) and by the density of clypeal punctures which are separated by a consistent 0.5–1 puncture diameters over the entire surface of the clypeus. Some individual species can also be separated with additional characters, as *A.baiocchii* has a small body size of 7–8 mm (usually 8–9 mm in *A.aerinifrons*), the propodeal triangle has a network of finely raised rugae covering ¾ of its surface (Fig. 4E; surface smooth in *A.aerinifrons*), and the tarsi are dark and the terga are completely impunctate (with the tarsi lightened orange and the terga with shallow but distinct punctures in *A.purpureomicans*). The male is unknown, but is expected to share the relatively elongate head which is unusual within this group of species.

##### Description.

**Female. *Body length***: 7–8 mm (Fig. 4A). ***Head***: Dark, 1.1× wider than long (Fig. 4B). Clypeus domed, variably sculptured, in basal ½ with fine granular shagreen, weakly shining, in apical ½ smooth and shining, polished; surface punctate, punctures separated by 1–3 puncture diameters (Fig. 4C). Process of labrum small, rounded trapezoidal, 2× wider than long, apical margin weakly emarginate. Gena marginally exceeding diameter of compound eye; ocelloccipital distance equals ½ diameter of lateral ocellus. Foveae dorsally occupying slightly < ½ space between compound eye and lateral ocellus, slightly narrowing ventrally, separated from inner margin of compound eye by distance subequal to its own diameter; foveae filled with brown hairs. Face, gena, vertex, and scape with sparse light brown hairs, none equalling length of scape. Antennae basally dark, A5–12 ventrally lightened by presence of grey-orange scales; A3 slightly exceeding A4+5, slightly shorter than A4+5+6.

***Mesosoma***: Scutum and scutellum with dense fine granular microreticulation, weakly shining to dull, laterally and anteriorly with weak but distinct green-purple metallic reflections; surface irregularly and obscurely punctate, punctures separated laterally by 1–3 puncture diameters, medially by 3–5 puncture diameters (Fig. 4D). Pronotum with strong humeral angle. Mesepisternum and dorsolateral parts of propodeum with dense granular microreticulation, dull; propodeal triangle delineated by change in surface sculpture, predominantly covered with finely raised rugae over ¾ of its surface (Fig. 4E). Mesosoma with light brown hairs, longest on mesepisternum, not equalling length of scape. Propodeal corbicula incomplete, dorsal fringe composed of long plumose light brown hairs, internal surface with scattered light brown hairs. Legs predominantly dark, apical tarsal segments lightened brownish, pubescence light brown. Flocculus complete but relatively sparse, composed of light brown plumose hairs; femoral and tibial scopae composed of light brown-golden simple hairs. Hind tarsal claws with very small inner tooth. Wings hyaline, stigma bright orange, venation dark orange, nervulus antefurcal.

***Metasoma***: Terga dark, apical margins weakly but distinctly depressed, partially lightened brown; tergal discs with regular granular microreticulation, weakly shining, essentially impunctate, with very weak and sparse punctures disappearing into background sculpture (Fig. 4F). Terga with scattered short white hairs, not forming apical hairbands. Apical fringe of T5 and hairs flanking pygidial plate orange. Pygidial plate large, broadly rounded triangular, lateral margin weakly raised and impunctate, internal surface densely punctate, punctures separated by < 0.5 puncture diameters.

**Male.** Unknown.

##### Etymology.

The species is named after Daniele Baiocchi who has collected insects across much of the Mediterranean basin and Middle East, and who led bee collection during the expedition to Duhok Governorate in May 2023.

##### Distribution.

Iraq (Kurdistan region).

#### ﻿11. Andrena (Plastandrena) bimaculata

Taxon classificationAnimaliaHymenopteraAndrenidae

(Kirby, 1802)

4F5ED72B-9293-54AA-B381-FC60EE63EFD5

##### Literature records.

Gusenleitner and Schwarz (2002: dot map 55); Scheuchl and Willner (2016); Ascher and Pickering (2023).

##### Remarks.

We have not examined any specimens of this species, but the presence of this species in Iraq is highly plausible based on its known global distribution, the map records indicated by Gusenleitner and Schwarz (2002), and the presence of this species in neighbouring Turkey and Iran. We note however that the species concept of *A.bimaculata* is unclear, and all Old World members of the subgenus Plastandrena require revision using molecular markers. It should therefore be considered in a sensu lato at the present time.

##### Distribution.

In a sensu lato, West and Central Palearctic to Mongolia (Gusenleitner and Schwarz 2002; Ascher and Pickering 2023).

#### ﻿12. Andrena (Cryptandrena) brumanensis

Taxon classificationAnimaliaHymenopteraAndrenidae

Friese, 1899 *

6E3BDEE0-7846-5BCC-AD5A-04CC67FE0CBE

##### Material examined.

**Iraq**: Duhok, Bessre [Besereh], Bablo, 36.8675°N, 43.1206°E, 1065 m, 5–6.v.2023, 1♀, leg. D. Baiocchi, MSVI; Duhok, E Mangesh, 37.0230°N, 43.1505°E, 1046 m, 8.v.2023, 1♂, leg. D. Baiocchi, MSVI.

##### Distribution.

Southern Europe to Turkey and the Near East, including Iraq* and Iran (Gusenleitner and Schwarz 2002; Wood and Monfared 2022).

#### ﻿13. Andrena (Truncandrena) caneae

Taxon classificationAnimaliaHymenopteraAndrenidae

Strand, 1915 *

A351C900-AA85-5947-AEF5-F283808E401D

##### Material examined.

**Iraq**: Duhok, Mt. Gara [S of Sarsing], 37.0158°N, 43.3506°E, 1912 m, 11.v.2023, 1♂, leg. D. Baiocchi, MSVI/TJWC.

##### Distribution.

Greece, Turkey, Cyprus, Syria, Iraq* (Gusenleitner and Schwarz 2002).

#### ﻿14. Andrena (Micrandrena) cedricola

Taxon classificationAnimaliaHymenopteraAndrenidae

Wood, 2020 *

34B7E17B-837F-54C8-AFB0-2473276DD97E

##### Material examined.

**Iraq**: Duhok, Mt. Gara [S of Sarsing], 37.0158°N, 43.3506°E, 1912 m, 11.v.2023, 2♂, leg. D. Baiocchi, MSVI/TJWC.

##### Distribution.

Israel, Lebanon, Syria, Turkey, Iraq* (Pisanty et al. 2022a).

#### ﻿15. Andrena (Chlorandrena) cinereophila

Taxon classificationAnimaliaHymenopteraAndrenidae

Warncke, 1965 *

A8FF93BC-E014-5BF3-A9F2-7EBE85AD51E2

##### Material examined.

**Iraq**: Duhok, Mt. Gara [S of Sarsing], 37.0158°N, 43.3506°E, 1912 m, 11.v.2023, 1♂, leg. D. Baiocchi, MSVI; **Lebanon**: Balbek-Hermel, Sefri, Haouch Snaid, AUB farm, 33.9244°N, 36.0754°E, 1000 m, 6.iv.2023, 11♀, leg. T.J. Wood, TJWC; Beqaa, Anjar, 1 km E, Armenian Cemetary, 33.7372°N, 35.9503°E, 900 m, 7.iv.2023, 1♀, leg. T.J. Wood, TJWC; Beqaa, Beqaa valley, Mansourah, Aammiq wetland preserve, 33.7321°N, 35.7853°E, 850 m, 3.iv.2023, 1♂, leg. T.J. Wood, TJWC; Beqaa, Beqaa valley, Qaraoun dam, 33.5483°N, 35.6851°E, 850 m, 6.iv.2023, 3♂, leg. T.J. Wood, TJWC.

##### Distribution.

East Mediterranean to Central Asia, including Lebanon* and Iraq* (Gusenleitner and Schwarz 2002; Osytshnjuk et al. 2005; Wood and Monfared 2022).

#### ﻿16. Andrena (Cordandrena) cordialis

Taxon classificationAnimaliaHymenopteraAndrenidae

Morawitz, 1878

D5E7FC41-F9BC-5FE7-86C2-3A5AF5E7E8B5

##### Literature records.

Morice (1921b); Derwesh (1965), mentioning “Survey of Iraq Fauna 1915–1919”; Augul (2018).

##### Material examined.

**Iraq**: Baiji [Saladin Governorate, 35.0299°N, 43.4489°E], 1–31.iii.1986, 1♀, leg. M. Carl, OÖLM; Baiji, 1–30.iv.1986, 2♂, leg. M. Carl, OÖLM.

##### Remarks.

*Andrenacordialis* is part of a tricky group of species to identify, with the species *A.cypria* Pittioni, 1950 and *A.torda* Warncke, 1965 described after the 1915–1919 survey was conducted. Morice (1921b) specifically mentioned the species from Basrah in southern Iraq. Based on our examined material, we can confidently confirm both *A.cordialis* and *A.torda* from Iraq, and so the record of Morice (1921b) is considered plausible. We note here that the “Survey of Iraq Fauna 1915–1919” is represented by the publications of Morice (1921a; 1921b), only Morice (1921b) dealing with the *Andrena* fauna.

##### Distribution.

Eastern Europe through Turkey and the Caucasus to Central Asia, including Iraq and Iran (Gusenleitner and Schwarz 2002; Osytshnjuk et al. 2005; Wood and Monfared 2022)

#### ﻿17. Andrena (Poecilandrena) crassana

Taxon classificationAnimaliaHymenopteraAndrenidae

Warncke, 1965 *

760A0EBA-60C9-5A40-8FB4-1D379663757E

##### Material examined.

**Iraq**: Duhok, Mt. Gara [S of Sarsing], 37.0158°N, 43.3506°E, 1912 m, 11.v.2023, 2♂, leg. D. Baiocchi, MSVI; **Lebanon**: Beqaa, Beqaa valley, Qaraoun, 3.5 km W of Madjal Balhis, 33.5377°N, 35.7038°E, 900 m, 4.iv.2023, 1♂, leg. T.J. Wood, TJWC.

##### Remarks.

Specimens from Iraq nominally belong to *A.crassana* s. str. And the specimen from Lebanon nominally belongs to ssp. *Inka* Warncke, 1969 (Levant). It is not clear if *inka* merits subspecific status.

##### Distribution.

*Andrenacrassana* sensu lato has a distribution of North Macedonia, Greece, Turkey, Cyprus, Israel and West Bank, Lebanon*, Jordan, Syria, Iraq*, Iran (Gusenleitner and Schwarz 2002; Pisanty et al. 2018; Wood and Monfared 2022).

#### ﻿18. Andrena (Aciandrena) curviocciput

Taxon classificationAnimaliaHymenopteraAndrenidae

Pisanty & Wood, 2022 *

33F66902-0824-55E5-984A-3DB6392A0CE8

##### Material examined.

**Iraq**: Duhok, Mt. Gara [S of Sarsing], 37.0158°N, 43.3506°E, 1912 m, 11.v.2023, 12♂, leg. D. Baiocchi, MSVI/TJWC.

##### Remarks.

Known from the Tanin-Tanin pass in south-eastern Turkey (Pisanty et al. 2022a), so its presence in northern Iraq was expected.

##### Distribution.

Israel, Lebanon, Turkey, Iraq* (Pisanty et al. 2022a).

#### ﻿19. Andrena

Taxon classificationAnimaliaHymenopteraAndrenidae

(incertae sedis) discordia Wood, 2023 *

4CECECFF-9220-5431-B531-01D70BAADAEA

##### Material examined.

**Iraq**: Duhok, Mt. Gara [S of Sarsing], 37.0158°N, 43.3506°E, 1912 m, 11.v.2023, 1♂, leg. D. Baiocchi, MSVI.

##### Distribution.

Turkey, Iraq*, Iran (Wood 2023).

#### ﻿20. Andrena (Aciandrena) duhokensis

Taxon classificationAnimaliaHymenopteraAndrenidae

Wood, sp. nov. *

64180BEE-5A83-55D4-A308-9FA3C2E06A0F

https://zoobank.org/3C35CEE1-7063-4C23-8577-1BA21A6CCAFE

##### Material examined.

***Holotype*: Iraq**: Duhok, Mt. Gara [S of Sarsing], 37.0158°N, 43.3506°E, 1912 m, 11.v.2023, 1♂, leg. D. Baiocchi, RMNH. ***Paratypes*: Iraq**: Same information as holotype, 7♂, MSVI/RMNH/TJWC/DUMAI.

##### Diagnosis.

**Figure 5.**
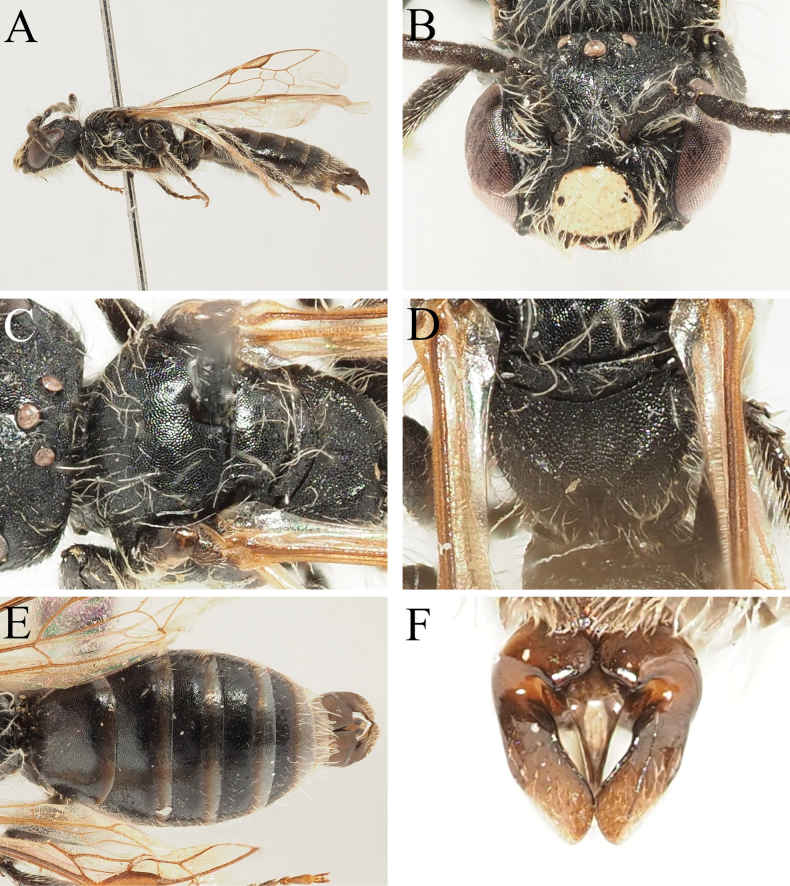
Andrena (Aciandrena) duhokensis sp. nov. male **A** habitus, lateral view **B** face, frontal view **C** scutum, dorsal view **D** propodeum, dorsal view **E** terga, dorsal view **F** genital capsule, dorsal view.

*Andrenaduhokensis* can be recognised as part of the subgenus Aciandrena Warncke, 1968 due to the small body size, dark integument with the exception of the pale-marked clypeus (Fig. 5A), finely shagreened propodeal triangle (Fig. 5D; without lateral or basal rugae), more or less impunctate terga (Fig. 5E), strongly antefurcal nervulus, and typical simple genital capsule (Fig. 5F; e.g., without completely reduced gonocoxae as in most members of the subgenus Graecandrena Warncke, 1968). Identification of *Aciandrena* species is extremely challenging due to the large number of often cryptic species found in dry environments, their often very local distributions, and lack of good characters in the female sex. Fortunately, males are a little easier to identify through the examination of the genital capsule. *Andrenaduhokensis* can be initially recognised due to its almost entirely yellow-marked clypeus (with the exception of two small black marks [sometimes absent] and some narrow black areas at the edges of the clypeus), placing it closest to *A.tenuis* Morawitz, 1877 which was described from the Caucasus. However, *A.duhokensis* can be recognised due to the unique form of the genital capsule which has the gonocoxal teeth with their apexes strongly truncate and asymmetrical so that they diverge from each other (Fig. 5F); in other words, they form somewhat pointed but flattened and diverging teeth. In comparative species of *Aciandrena*, the gonocoxal teeth are either strongly produced and truncate (e.g., *A.deminuta* Wood 2022 from Iran), produced into narrowly pointed or more broadly rounded teeth (e.g., *A.tenuis* from Turkey and the Caucasus, *A.aciculata* Morawitz, 1886 from Europe to the Caucasus), or reduced and truncate but never forming diverging teeth (e.g., *A.judaea* Scheuchl & Pisanty, 2016 from Israel). In this context, *A.duhokensis* can be separated from all similar species.

##### Description.

**Female.** Unknown.

**Male.** Body length 4–5 mm (Fig. 5A). ***Head***: Dark, 1.25× wider than long (Fig. 5B). Clypeus weakly domed, predominantly yellow or pale yellow marked with exception of two black dots (sometimes absent) and narrow black markings laterally; surface finely shagreened and weakly shining, irregularly punctate, punctures separated by 1–3 puncture diameters. Process of labrum small, rounded trapezoidal, 2× wider than long, apical margin weakly emarginate. Gena subequal to diameter of compound eye; ocelloccipital distance equals diameter of lateral ocellus. Head covered with dirty white hairs, none equalling length of scape. Antennae basally dark, A4–13 ventrally lightened by presence of grey scales; A3 exceeding length of A4, shorter than A4+5; A4 sub-square, shorter than long, A5 quadrate, as long as broad.

***Mesosoma***: Scutum and scutellum with fine granular shagreen, weakly shining, irregularly punctate, punctures separated by 1–4 puncture diameters (Fig. 5C). Mesepisternum and dorsolateral parts of propodeum with regular but large microreticulation, dull to weakly shining; propodeal triangle large, indicated by change in surface sculpture, internal surface with fine granular microreticulation, in some parts with weakly raised network of carinae between microreticulation, surface dull (Fig. 5D). Mesosoma with sparse short whitish hairs. Legs predominantly dark, apical tarsal segments slightly lightened orange, pubescence white. Hind tarsal claws with strong inner tooth. Wings hyaline, stigma, and venation pale orange, nervulus very strongly antefurcal.

***Metasoma***: Terga predominantly dark, tergal margins weakly but distinctly depressed, broadly lightened hyaline yellow-brown (Fig. 5E). Tergal discs finely microreticulate to shagreened, weakly shining, surface essentially impunctate, with scattered obscure punctures disappearing into underlying sculpture. Terga with sparse scattered short hairs, not forming hairbands. T6 and T7 with long light brown hairs overlying pseudopygidial plate of T7. S8 narrow, apex slightly broadened like a fish-tail, truncate, ventral surface with dense lateral fan of brown hairs. Genital capsule compact, gonocoxae produced into apical teeth, teeth strongly truncate, apexes angled and diverging from each other to form angular anteriorly projecting teeth (Fig. 5F). Gonostyli flattened and spatulate, internal margins raised, forming slight bump on inner margin basally. Penis valves moderate, occupying ½ space between gonostyli basally, strongly tapering apically.

##### Etymology.

The name is derived from the city of Duhok which gives its name to the province in which these specimens were collected.

##### Distribution.

Iraq (Kurdistan region).

#### ﻿21. Andrena (Micrandrena) elam

Taxon classificationAnimaliaHymenopteraAndrenidae

Wood, 2022 *

BA4086AE-5DC1-5BE1-9473-7533CC071653

##### Material examined.

**Iraq**: Duhok, Mt. Gara [S of Sarsing], 37.0158°N, 43.3506°E, 1912 m, 11.v.2023, 23♂, 1♀, leg. D. Baiocchi, MSVI/TJWC.

##### Remarks and diagnosis.

**Figure 6.**
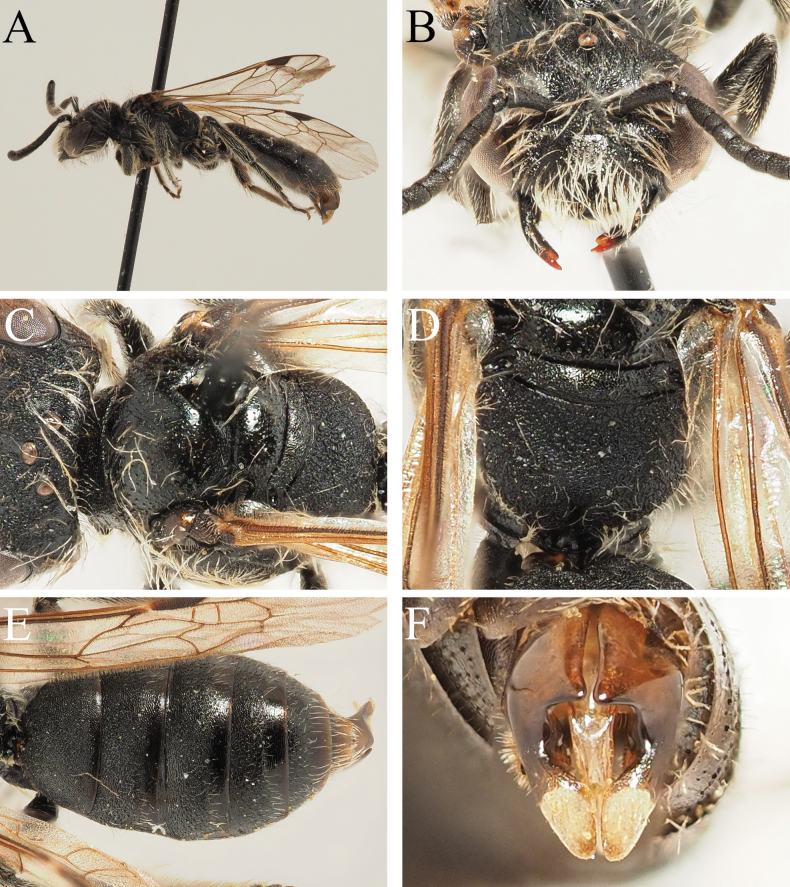
Andrena (Micrandrena) elam Wood, 2022 male **A** habitus, lateral view **B** face, frontal view **C** scutum, dorsal view **D** propodeum, dorsal view **E** terga, dorsal view **F** genital capsule, dorsal view.

Wood in Wood and Monfared (2022) described *A.elam* in the female sex from western and southern Iran, and Wood (2023) reported further specimens from southern and south-eastern Turkey. Numerous male specimens from northern Iraq are now available. They can be recognised as *Micrandrena* due to the small body size, dark integument (including clypeus Fig. 6B), entirely rugose propodeal triangle (Fig. 6D), and normal genital capsule. As in the female sex, they can be separated from other *Micrandrena* species by the combination of smooth and shining scutum and scutellum with moderately dense punctures (Fig. 6C), the tergal discs with extremely strong and dense reticulation (Fig. 6E), this reticulation becoming much weaker on the marginal areas, with punctures visible on the discs of T3–4 (though comparatively weaker and less visible than in the female), and by the tergal margins becoming progressively more strongly depressed. Additionally, the genital capsule is distinctive (Fig. 6F), somewhat elongate, with the gonocoxae produced into weak angular teeth, with thickened gonostyli with a weak rounded bump on their inner margins and outer surface lightened and covered with short golden hairs. It resembles the form of two former *Fumandrena* (= *Micrandrena*) species (*A.fabrella* Pérez, 1895 (western Mediterranean) and *A.tomora* Warncke, 1975 (eastern Mediterranean)), but these have the gonostyli apically flattened, not thickened. It is very close to *A.subviridula* Wood, 2022 (northern Iran, see illustrations in Wood and Monfared 2022), but can be separated by the gonocoxae produced into apical points (in *A.subviridula* with the gonocoxae apically truncate), by the weaker bump on the inner margins of the gonostyli (in *A.subviridula* with this bump comparatively more pronounced), and by the more extensively shiny scutum which lacks subtle greasy green-metallic reflections (in *A.subviridula* with the scutum more extensively shagreened anteriorly and laterally).

##### Description.

**Male. *Body length***: 6 mm (Fig. 6A). ***Head***: Dark, 1.45× wider than long (Fig. 6B). Clypeus domed, densely punctate, punctures separated by < 0.5–1 puncture diameter, surface with weakly raised latitudinal striations between punctures, surface weakly shining. Process of labrum trapezoidal, 2× wider than long, anterior margin weakly emarginate, surface smooth and shining. Gena equalling diameter of compound eye; ocelloccipital distance equalling diameter of lateral ocellus. Head with sparse whitish hairs, none equalling length of scape; clypeus in fresh individuals with moderate “beard” of ventrally projecting pale hairs. Antennae dark, A5–13 ventrally lightened by presence of silver-grey scales; A3 exceeding length of A4, shorter than A4+5.

***Mesosoma***: Scutum and scutellum polished and shining over almost entire surface, scutum weakly shagreened anteriorly; irregularly punctate, punctures separated by 1–3 puncture diameters (Fig. 6C). Pronotum rounded. Mesepisternum microreticulate, dull. Dorsolateral parts of propodeum microreticulate, dull, sculpture overlain by network of irregular raised rugosity; propodeal triangle laterally defined by irregular carinae, internal surface densely covered with network of evenly-spaced rugae, propodeal triangle thus well-differentiated from dorsolateral parts of propodeum (Fig. 6D). Mesosoma covered with sparse light brown hairs, none equalling length of scape. Legs dark, apical tarsal segments paler dark brown, pubescence whitish. Hind tarsal claws with inner tooth. Wings hyaline, stigma dark brown, venation orange-brown, nervulus interstitial.

***Metasoma***: Tergal discs dark, marginal areas with apical rim narrowly lightened brown (Fig. 6E). Tergal discs strongly reticulate, reticulation strongest on T1, surface dull, becoming progressively weaker on subsequent terga, discs of T5 and T6 weakly shining; marginal areas shagreened, weakly shining. Tergal discs obscurely punctate, T1 almost impunctate, punctures progressively more visible on subsequent terga, punctures on disc of T4 separated by 1–2 puncture diameters. Tergal discs with scattered pale hairs, not forming hairbands. T6 and T7 with light brown hairs overlying pseudopygidial plate. S8 columnar, apical margin truncate, ventral surface with sparse short brown hairs. Genital capsule somewhat elongate, gonocoxae with apical margins produced into slightly projecting narrow apical teeth (Fig. 6F). Gonostyli robust, thickened, with weak bump on internal margins basally, apically weakly flattened, apical ½ lightened hyaline yellowish. Penis valves narrow, occupying ½ space between gonostyli, uniformly narrowing towards apex.

##### Distribution.

Southern and south-eastern Turkey, northern Iraq*, and western and southern Iran (Wood and Monfared 2022; Wood 2023).

#### ﻿22. Andrena (Lepidandrena) elisaria

Taxon classificationAnimaliaHymenopteraAndrenidae

Gusenleitner, 1998 *

67CC5BAC-B278-5740-A073-5FCD0DD04123

##### Material examined.

**Iraq**: Duhok, Mt. Gara [S of Sarsing], 37.0158°N, 43.3506°E, 1912 m, 11.v.2023, 1♂, leg. D. Baiocchi, MSVI.

##### Distribution.

Israel, Turkey, Iraq*, Iran (Gusenleitner 1998; Pisanty et al. 2018; Wood and Monfared 2022).

#### ﻿23. Andrena (incertae sedis) euzona

Taxon classificationAnimaliaHymenopteraAndrenidae

Pérez, 1895

DC5495CC-CD34-5497-A9E3-1D103BF3F38A

##### Literature records.

Wood and Monfared (2022).

##### Distribution.

Morocco, Algeria, Tunisia, Libya, Egypt, Israel and West Bank, Jordan, Syria, Iraq, Iran (Gusenleitner and Schwarz 2002; Wood and Monfared 2022).

#### ﻿24. Andrena (Plastandrena) eversmanni

Taxon classificationAnimaliaHymenopteraAndrenidae

Radoszkowski, 1867

A884EC9D-9B84-5669-A14A-B37C380AAB7B

##### Literature records.

Wood and Monfared (2022).

##### Distribution.

Turkey, Armenia, Iraq, Iran, Russia (European part), Turkmenistan, Uzbekistan, Kazakhstan, Tajikistan, Pakistan, Mongolia, China (Wood and Monfared 2022; Ascher and Pickering 2023).

#### ﻿25. Andrena (Hoplandrena) ferox

Taxon classificationAnimaliaHymenopteraAndrenidae

Smith, 1847 *

7C2998DC-30A6-5C89-901E-8A1087254B44

##### Material examined.

**Iraq**: Duhok, Mt. Gara [S of Sarsing], 37.0158°N, 43.3506°E, 1912 m, 11.v.2023, 1♀, leg. D. Baiocchi, MSVI; **Lebanon**: Beqaa, Beqaa valley, Mansourah, Aammiq wetland preserve, 33.7321°N, 35.7853°E, 850 m, 3.iv.2023, 1♀, leg. T.J. Wood, TJWC.

##### Distribution.

West Palaearctic to the Middle East, including Lebanon*, Iraq*, Iran (Gusenleitner and Schwarz 2002; Scheuchl and Willner 2016; Wood and Monfared 2022).

#### ﻿26. Andrena (Melandrena) flavipes

Taxon classificationAnimaliaHymenopteraAndrenidae

Panzer, 1799

E444AC44-1A14-57BB-967B-39C2B19F67DD

##### Literature records.

Morice (1921b); Derwesh (1965), mentioning “Survey of Iraq Fauna 1915–1919”; Gusenleitner and Schwarz (2002: dot map 161); Scheuchl and Willner (2016); Augul (2018); Ascher and Pickering (2023).

##### Remarks.

We have not examined any specimens of this species, but the presence of this species in Iraq is extremely plausible based on its known global distribution, its abundance in a wide variety of habitats, the map records indicated by Gusenleitner and Schwarz (2002), and the presence of this species in neighbouring Turkey and Iran.

##### Distribution.

West and Central Palearctic (Gusenleitner and Schwarz 2002; Ascher and Pickering 2023).

#### ﻿27. Andrena (Bryandrena) florea

Taxon classificationAnimaliaHymenopteraAndrenidae

Fabricius, 1793 *

51175E8E-CED2-50B8-A61A-D4049DF65DF8

##### Material examined.

**Iraq**: Duhok, Mt. Gara [S of Sarsing], 37.0158°N, 43.3506°E, 1912 m, 11.v.2023, 1♂, leg. D. Baiocchi, MSVI.

##### Distribution.

West Palaearctic, from Morocco to Iraq*, Iran, Turkmenistan, and the Ural mountains (Gusenleitner and Schwarz 2002).

#### ﻿28. Andrena (Ulandrena) fulvitarsis

Taxon classificationAnimaliaHymenopteraAndrenidae

Brullé, 1832

6EC0CFAC-4EBA-506C-BAF9-399C44E815E3

##### Literature records.

Gusenleitner and Schwarz (2002: dot map 177); Ascher and Pickering (2023).

##### Remarks.

We have not examined any specimens of this species, but the presence of this species in Iraq is plausible based on its known global distribution, the map records indicated by Gusenleitner and Schwarz (2002), and the presence of this species in neighbouring Turkey.

##### Distribution.

East Mediterranean, from Italy to Crimea, Turkey, the Levant, Iraq (Gusenleitner and Schwarz 2002).

#### ﻿29. Andrena (incertae sedis) garrula

Taxon classificationAnimaliaHymenopteraAndrenidae

Warncke, 1965 *

AF06F9E2-7B7D-5CA6-9AFD-3DF45DA31F61

##### Material examined.

**Iraq**: Duhok, Mt. Gara [S of Sarsing], 37.0158°N, 43.3506°E, 1912 m, 11.v.2023, 1♀, leg. D. Baiocchi, MSVI.

##### Remarks.

This specimen appears to be *A.garrula* s. str. as opposed to the Levantine ssp. lomvia Warncke, 1969; the difference between the two subspecies is difficult to distinguish in the female sex.

##### Distribution.

Bulgaria, Turkey, Israel and West Bank, Lebanon, Jordan, Syria, Iraq*, Iran (Gusenleitner and Schwarz 2002; Wood et al. 2020; Wood and Monfared 2022).

#### ﻿30. Andrena (Melandrena) grandilabris

Taxon classificationAnimaliaHymenopteraAndrenidae

Pérez, 1903 *

4A1386C5-D55B-552E-98E4-B91D59D88F2E

##### Material examined.

**Iraq**: Duhok, Mt. Gara [S of Sarsing], 37.0158°N, 43.3506°E, 1912 m, 11.v.2023, 4♂, leg. D. Baiocchi, MSVI.

##### Remarks.

Turkey, Iraq*, Iran (Wood and Monfared 2022).

#### ﻿31. Andrena (Euandrena) hermonella

Taxon classificationAnimaliaHymenopteraAndrenidae

Scheuchl & Pisanty, 2016 *

3D962AF5-130A-5A24-9F3F-76C24098B3A0

##### Material examined.

**Iraq**: Duhok, Mt. Gara [S of Sarsing], 37.0158°N, 43.3506°E, 1912 m, 11.v.2023, 2♀, leg. D. Baiocchi, MSVI.

##### Remarks.

The original female specimens described by Pisanty et al. (2016) actually belonged to *A.gageae* Wood & Pisanty, 2022 (see Pisanty et al. 2022a). The true female of *A.hermonella* is being described from south-eastern Turkey (Wood, in press). This extends the range from south-eastern Turkey into northern Iraq.

##### Distribution.

Israel, Turkey, Iraq* (Pisanty et al. 2016; Wood, unpublished data).

#### ﻿32. Andrena

Taxon classificationAnimaliaHymenopteraAndrenidae

(incertae sedis) hosseiniiae Wood & Monfared, 2022 *

B797B06F-C7A5-5151-9A7C-8634D4622F3D

##### Material examined.

**Iraq**: Duhok, Mt. Gara [S of Sarsing], 37.0158°N, 43.3506°E, 1912 m, 11.v.2023, 1♀, leg. D. Baiocchi, MSVI.

##### Remarks.

The first record of this species outside of Iran, with all previous records coming from close to Yasuj (Kohgiluyeh and Boyer-Ahmad Province) in southern Iran.

##### Distribution.

Iraq* and Iran (Wood and Monfared 2022).

#### ﻿33. Andrena (Poecilandrena) hybrida

Taxon classificationAnimaliaHymenopteraAndrenidae

Warncke, 1975 *

54955105-428A-5ABC-8CD2-01E3F2EA37B2

##### Material examined.

**Iraq**: Duhok, Mt. Gara [S of Sarsing], 37.0158°N, 43.3506°E, 1912 m, 11.v.2023, 1♀, leg. D. Baiocchi, MSVI.

##### Remarks.

Multiple subspecies are described for *A.hybrida*; their statuses require further study, but this is limited by the scarcity of material. Iraqi material conforms to *A.hybrida* s. str.

##### Distribution.

Ukraine, Russia (European part), Turkey, Iraq*, Iran (Gusenleitner and Schwarz 2002; Wood and Monfared 2022).

#### ﻿34. Andrena (Graecandrena) hyemala

Taxon classificationAnimaliaHymenopteraAndrenidae

Warncke, 1973 *

296CEFE4-59D5-5F5D-BDE5-B23C93DF468E

##### Material examined.

**Iraq**: Duhok, Mt. Gara [S of Sarsing], 37.0158°N, 43.3506°E, 1912 m, 11.v.2023, 7♂, leg. D. Baiocchi, MSVI; Mosul, edges of a river, 7.iv.1988, 3♀, leg. Olejníček, OÖLM.

##### Remarks.

The specimens presented here appear to be A.hyemala s. str. The statuses of the subspecies repressa Warncke, 1975 (Levant) and *kushika* Osytshnjuk, 1994 (Central Asia) require further study.

##### Distribution.

*Andrenahyemala* sensu lato is distributed from south-eastern Europe through the Middle East (including Iraq* and Iran) to Central Asia (Gusenleitner and Schwarz 2002; Osytshnjuk et al. 2008; Wood and Monfared 2022).

#### ﻿35. Andrena (Ulandrena) kriechbaumeri

Taxon classificationAnimaliaHymenopteraAndrenidae

Schmiedeknecht, 1883 *

F6124294-B9AF-52AD-A4B0-6B4F38113417

##### Material examined.

**Iraq**: Duhok, E Mangesh, 37.0230°N, 43.1505°E, 1046 m, 8.v.2023, 1♂, leg. D. Baiocchi, MSVI; **Syria**: Apamea, 65 km NW Hama, 270 m, 18.iv.1992, 1♂, leg. K. Warncke, OÖLM; **Turkey**: Siirt, 5 km E Eruh, 1000 m, 26.v.1983, 1♂, leg. K. Warncke, OÖLM.

##### Remarks.

The finding of *A.kriechbaumeri* in Iraq is notable. One of the most common and abundant *Andrena* species in the southern Balkans, *A.kriechbaumeri* is much less common in Turkey where it is largely restricted to western Turkey (see distribution map of Gusenleitner and Schwarz 2002). Examination of undetermined material in the OÖLM collection produced specimens from northern Syria and a specimen from near to Siirt in eastern Turkey. To our knowledge, this is the most easterly specimen known from Turkey. In this context, the presence of *A.kriechbaumeri* in northern Iraq represents less of an outlier, though it is clear that *A.kriechbaumeri* has a population centre in the Balkan Peninsula.

##### Distribution.

Europe from Italy to Turkey, Syria*, and Iraq* (Gusenleitner and Schwarz 2002).

#### ﻿36. Andrena (Poecilandrena) laticeps

Taxon classificationAnimaliaHymenopteraAndrenidae

Morawitz, 1878 *

43BE371E-403D-57D1-B9DC-1011F7321733

##### Material examined.

**Iraq**: Duhok, Mt. Gara [S of Sarsing], 37.0158°N, 43.3506°E, 1912 m, 11.v.2023, 3♂, leg. D. Baiocchi, MSVI.

##### Distribution.

Turkey, Georgia, Armenia, Iraq*, Iran (Wood and Monfared 2022; Ascher and Pickering 2023).

#### ﻿37. Andrena (Melandrena) limata

Taxon classificationAnimaliaHymenopteraAndrenidae

Smith, 1853 *

68794679-3F21-5014-87F9-1BC2D6D9ECA4

##### Material examined.

**Iraq**: Baiji [Saladin Governorate, 35.0299°N, 43.4489°E], 1–31.iii.1986, 3♀, leg. M. Carl, OÖLM.

##### Distribution.

West and Central Palearctic including Iraq* and Iran (Gusenleitner and Schwarz 2002; Osytshnjuk et al. 2008; Wood and Monfared 2022).

#### ﻿38. Andrena (Micrandrena) luscinia

Taxon classificationAnimaliaHymenopteraAndrenidae

Warncke, 1975 *

3AE2497A-4F68-5DB8-9374-95A189F6B5C7

##### Material examined.

**Iraq**: Duhok, Mt. Gara [S of Sarsing], 37.0158°N, 43.3506°E, 1912 m, 11.v.2023, 1♀, leg. D. Baiocchi, MSVI; **Lebanon**: Beqaa, Rachaiya, 5 km S, Mount Hermon nature reserve, 33.4586°N, 35.8395°E, 1500 m, 8.iv.2023, 2♀, leg. T.J. Wood, TJWC.

##### Distribution.

Israel, Lebanon*, Turkey, Iraq*, Iran (Pisanty et al. 2018; Wood and Monfared 2022).

#### ﻿39. Andrena

Taxon classificationAnimaliaHymenopteraAndrenidae

(incertae sedis) monacha Warncke, 1965 *

DF20AAC7-F845-58B8-9B40-ECCD4739E9A9

##### Material examined.

**Iraq**: Duhok, Mt. Gara [S of Sarsing], 37.0158°N, 43.3506°E, 1912 m, 11.v.2023, 1♂, leg. D. Baiocchi, MSVI.

##### Distribution.

Greece, Turkey, Cyprus, Lebanon, Syria, Iraq*, Iran (Gusenleitner and Schwarz 2002; Wood et al. 2020; Wood and Monfared 2022).

#### ﻿40. Andrena (Melandrena) morio

Taxon classificationAnimaliaHymenopteraAndrenidae

Brullé, 1832

90A4BDB0-655D-5A74-96AC-76C25D73CBA0

##### Literature records.

Khalaf and Al-Omar (1974); Gusenleitner and Schwarz (2002: dot map 310); Scheuchl and Willner (2016); Augul (2018).

##### Remarks.

We have not examined any specimens of this species, but the presence of this species in Iraq is plausible based on its known global distribution, the map records indicated by Gusenleitner and Schwarz (2002), and the presence of this species in neighbouring Turkey and Iran.

##### Distribution.

West and Central Palaearctic (Gusenleitner and Schwarz 2002; Osytshnjuk et al. 2008; Wood and Monfared 2022).

#### ﻿41. Andrena (Melandrena) nitidemula

Taxon classificationAnimaliaHymenopteraAndrenidae

Scheuchl & Hazir, 2012 *

9A90C1C4-EE1F-5E30-A34F-E63D893CD7F6

##### Material examined.

**Iraq**: Duhok, Mt. Gara [S of Sarsing], 37.0158°N, 43.3506°E, 1912 m, 11.v.2023, 1♀, leg. D. Baiocchi, MSVI.

##### Distribution.

Greece, Turkey, Syria, Georgia, Armenia, Iraq*, Iran (Wood and Monfared 2022).

#### ﻿42. Andrena (Micrandrena) obsidiana

Taxon classificationAnimaliaHymenopteraAndrenidae

Wood, 2022 *

AA082101-CC64-5619-B49C-AAD0DDBC8695

##### Material examined.

**Iraq**: Duhok, Mt. Gara [S of Sarsing], 37.0158°N, 43.3506°E, 1912 m, 11.v.2023, 15♂, 4♀, leg. D. Baiocchi, MSVI/TJWC; **Turkey**: Hakkâri, pass E of Uludere, 6.vi.1977, 1♂, 2♀, leg. K. Warncke, OÖLM/TJWC; Hakkâri, Tanin-Tanin-Pass, 2500 m, 2.vi.1980, 3♂, 1♀, leg. K. Warncke, OÖLM.

##### Remarks and diagnosis.

Wood and Monfared (2022) described *A.obsidiana* in the female sex from southern and south-eastern Turkey and western and southern Iran. Numerous male specimens from northern Iraq are now available, as well as some additional specimens from south-eastern Turkey that were not available for study at the time of the original description. One of these *A.obsidiana* specimens from Turkey (a female) was separated by Warncke and labelled as “*A.ferulella* spec. nov.”, but this name was never published.

**Figure 7.**
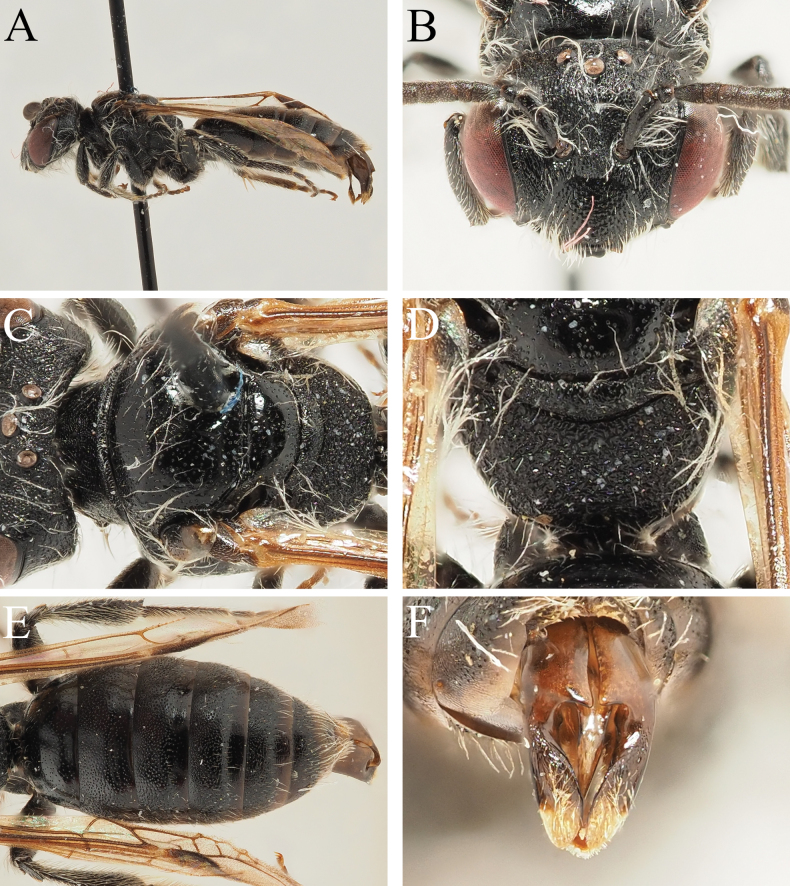
Andrena (Micrandrena) obsidiana Wood, 2022 male **A** habitus, lateral view **B** face, frontal view **C** scutum, dorsal view **D** propodeum, dorsal view **E** terga, dorsal view **F** genital capsule, dorsal view.

*Andrenaobsidiana* can be recognised as *Micrandrena* due to the small body size, dark integument (including clypeus Fig. 7B), entirely rugose propodeal triangle (Fig. 7D), and normal genital capsule (Fig. 7F). Due to the smooth and shining scutum and scutellum with moderately dense punctures (Fig. 7C) and the genital capsule (gonocoxae produced into weak angular teeth, with thickened gonostyli with very small rounded bump on their inner margins, and outer surface lightened and covered with short golden hairs) it is close to *A.elam* and *A.subviridula*, but it can instantly be separated from them by the sculpture of the terga which is almost completely smooth and shining (Fig. 7E), with only superficial shagreenation at the base of the tergal discs. There are also differences in the genital capsule, with that of *A.obsidiana* being comparatively more elongate, with the bump on the inner margin of the gonostyli reduced to a relatively tiny projection.

##### Description.

**Male. *Body length***: 5–6 mm (Fig. 7A). ***Head***: Dark, 1.25× wider than long (Fig. 7B). Clypeus domed, densely punctate, punctures separated by < 0.5–1 puncture diameter, surface weakly shining. Process of labrum trapezoidal, 2× wider than long, anterior margin weakly emarginate, surface weakly shining. Gena equalling diameter of compound eye; ocelloccipital distance subequal to diameter of lateral ocellus. Head with sparse whitish hairs, none equalling length of scape; clypeus in fresh individuals with moderate “beard” of ventrally projecting pale hairs. Antennae basally dark, A5–13 ventrally lightened by presence of brownish-grey scales; A3 exceeding length of A4, slightly shorter than A4+5.

***Mesosoma***: Scutum and scutellum polished and shining over almost entire surface, scutum weakly shagreened anteriorly; irregularly punctate, punctures separated by 1–3 puncture diameters, slightly denser on scutum (Fig. 7C). Pronotum rounded. Mesepisternum microreticulate, dull. Dorsolateral parts of propodeum microreticulate, dull, sculpture overlain by dense network of raised rugosity; propodeal triangle poorly defined laterally, without clear linear carinae, internal surface densely covered with dense network of rugae, propodeal triangle thus poorly differentiated from dorsolateral parts of propodeum (Fig. 7D). Mesosoma covered with sparse whitish to light brown hairs, none equalling length of scape. Legs dark, apical tarsal segments paler dark brown, pubescence whitish. Hind tarsal claws with inner tooth. Wings hyaline, stigma dark brown, venation orange-brown, nervulus weakly to strongly antefurcal.

***Metasoma***: Tergal discs dark, marginal areas with apical rim obscurely lightened dark brown (Fig. 7E). Tergal discs with weak sculpture, T1 polished and shining, base of remaining terga with fine shagreen, otherwise smooth and shining. Terga deeply punctate, T1 with punctures separated by 1–2 puncture diameters, remaining tergal discs with punctures separated by 1 puncture diameter; punctures only partially extending onto marginal areas, becoming weaker on apical terga, marginal area of T5 almost impunctate. Tergal margins progressively more strongly depressed, weakly on T1, strongly on T5. Tergal discs with scattered pale hairs, not forming hairbands. T6 andT7 with light brown hairs overlying pseudopygidial plate. S8 columnar, apical margin rounded, ventral surface with dense fan of short brown hairs. Genital capsule somewhat elongate, gonocoxae with apical margins produced into slightly projecting narrow apical teeth (Fig. 7F). Gonostyli robust, thickened, with weak and obscure bump on internal margins basally, apical ½ lightened hyaline yellowish. Penis valves narrow, occupying ½ space between gonostyli, uniformly narrowing towards apex.

##### Distribution.

Southern and south-eastern Turkey, northern Iraq*, and western and southern Iran (Wood and Monfared 2022).

#### ﻿43. Andrena (Micrandrena) oedicnema

Taxon classificationAnimaliaHymenopteraAndrenidae

Warncke, 1975 *

2D403CEC-4159-57BB-8377-E80BB4258C54

##### Material examined.

**Iraq**: Duhok, Mt. Gara [S of Sarsing], 37.0158°N, 43.3506°E, 1912 m, 11.v.2023, 2♂, leg. D. Baiocchi, MSVI;

##### Distribution.

Greece, Turkey, Israel and West Bank, Lebanon, Jordan, Syria, Turkey, Iraq*, Iran (Gusenleitner and Schwarz 2002; Pisanty et al. 2018; Wood and Monfared 2022).

#### ﻿44. Andrena (Pallandrena) pallidicincta

Taxon classificationAnimaliaHymenopteraAndrenidae

Brullé, 1832 *

C826897E-DEAA-5FD5-A9F5-23FBB0ACC90F

##### Material examined.

**Iraq**: Duhok, Mt. Gara [S of Sarsing], 37.0158°N, 43.3506°E, 1912 m, 11.v.2023, 11♀, leg. D. Baiocchi, MSVI/TJWC.

##### Distribution.

South-eastern Europe to Turkey, Lebanon, Israel, and Iraq* (Gusenleitner and Schwarz 2002; Wood et al. 2020).

#### ﻿45. Andrena (Truncandrena) pareklisiae

Taxon classificationAnimaliaHymenopteraAndrenidae

Mavromoustakis, 1956 *

FE1B7251-2B43-5764-A127-8CB15AE87E9C

##### Material examined.

**Iraq**: Duhok, Mt. Gara [S of Sarsing], 37.0158°N, 43.3506°E, 1912 m, 11.v.2023, 6♀, leg. D. Baiocchi, MSVI/TJWC.

##### Distribution.

Cyprus, Turkey, Lebanon, Syria, Iraq*, Iran (Wood 2023).

#### ﻿46. Andrena (Truncandrena) rufomaculata

Taxon classificationAnimaliaHymenopteraAndrenidae

Friese, 1921 *

67584F7A-4607-5812-A672-225BC047B39F

##### Material examined.

**Iraq**: Duhok, Mt. Gara [S of Sarsing], 37.0158°N, 43.3506°E, 1912 m, 11.v.2023, 1♀, leg. D. Baiocchi, MSVI.

##### Distribution.

Turkey, Israel and West Bank, Lebanon, Jordan, Syria, Iraq*, Iran (Wood et al. 2020; Wood and Monfared 2022).

#### ﻿47. Andrena (Suandrena) savignyi

Taxon classificationAnimaliaHymenopteraAndrenidae

Spinola, 1838

16C0A155-88E2-5A75-AF3E-36323C9432F3

##### Literature records.

Moalif (1994); Ascher and Pickering (2023).

##### Remarks.

We have not examined any specimens of this species, but the listing is plausible due to the presence of multiple specimens of *A.savignyi* in western and southern Iran (Wood and Monfared 2022), and the preference of *A.savignyi* for dry desert and semi-desert habitats.

##### Distribution.

West and Central Palaearctic (Wood and Monfared 2022; Ascher and Pickering 2023).

#### ﻿48. Andrena (Micrandrena) sillatahistrionica

Taxon classificationAnimaliaHymenopteraAndrenidae

Warncke, 1975 *

61EC879C-62C9-5805-B0AB-CC3E7B38B99E

##### Material examined.

**Iraq**: Duhok, Mt. Gara [S of Sarsing], 37.0158°N, 43.3506°E, 1912 m, 11.v.2023, 4♂, 1♀, leg. D. Baiocchi, MSVI.

##### Distribution.

Subspecies histrionica is found in Turkey, Iraq*, and Iran only (Wood and Monfared 2022). It may merit species status due to differences in the male genital capsule, but genetic data are required.

#### ﻿49. Andrena (Euandrena) symphyti

Taxon classificationAnimaliaHymenopteraAndrenidae

Schmiedeknecht, 1883 *

09E7DB29-4894-5CA2-8A9D-84B070C26EF0

##### Material examined.

**Iraq**: Duhok, Mt. Gara [S of Sarsing], 37.0158°N, 43.3506°E, 1912 m, 11.v.2023, 5♂, 1♀, leg. D. Baiocchi, MSVI.

##### Distribution.

West Palaearctic, including Iraq* and Iran (Gusenleitner and Schwarz 2002; Wood and Monfared 2022).

#### ﻿50. Andrena (Chlorandrena) tadauchii

Taxon classificationAnimaliaHymenopteraAndrenidae

Gusenleitner, 1998 *

A8A568BC-1A4E-50AB-84B8-6F8DC20F4F01

##### Material examined.

**Iraq**: Duhok, E Mangesh, 37.0230°N, 43.1505°E, 1046 m, 8.v.2023, 1♀, leg. D. Baiocchi, MSVI; Duhok, Mt. Gara [S of Sarsing], 37.0158°N, 43.3506°E, 1912 m, 11.v.2023, 14♂, leg. D. Baiocchi, MSVI/TJWC; **Lebanon**: Balbek-Hermel, Sefri, Haouch Snaid, AUB farm, 33.9244°N, 36.0754°E, 1000 m, 6.iv.2023, 2♂, 20♀, leg. T.J. Wood, TJWC; Beqaa, Anjar, 1 km E, Armenian Cemetary, 33.7372°N, 35.9503°E, 900 m, 7.iv.2023, 1♀, leg. T.J. Wood, TJWC; Beqaa, Beqaa valley, Mansourah, Aammiq wetland preserve, 33.7321°N, 35.7853°E, 850 m, 3.iv.2023, 1♀, leg. T.J. Wood, TJWC; Bekaa, Qob Elias valley, 33.7989°N, 35.8192°E, 900 m, 5.iv.2023, 1♀, leg. T.J. Wood, TJWC; Hrar-Akkar, 34.4572°N, 36.1228°E, 900 m, 17.iv.2021, 1♂, leg. A. Saab, TJWC.

##### Remarks.

Confirmed as present in Lebanon after the unclear listing of Grace (2010).

##### Distribution.

Israel and West Bank, Lebanon*, Syria, Turkey, Iraq* (Pisanty et al. 2022a).

#### ﻿51. Andrena (Ulandrena) tadornacallida

Taxon classificationAnimaliaHymenopteraAndrenidae

Warncke, 1974 *

F49324B6-78FE-5A4C-87AB-3A19A46D1E87

##### Material examined.

**Iraq**: Hatra [Nineveh Governorate, 35.5759°N, 42.7254°E], 6.iv.1988, 1♂, leg. Olejníček, OÖLM; **Saudi Arabia**: Abha, 2000 m, 31.iii.1980, 1♂, leg. K.M. Guichard, NHMUK; As Nimas [Al Namas], 2450 m, 3–4.iv.1980, 3♂, 3♀, leg. K.M. Guichard, NHMUK; **Syria**: 110 km E of Palmyra, 350 m, 21.iv.1992, 2♀, leg. K. Warncke, OÖLM; 30 km W Palmyra, 580 m, 23.iv.1992, 6♀, leg. M. Kraus and K. Warncke, OÖLM; Suweidaono, ENE 80km, 700 m, 27.iii.1988, 2♂, leg. M. Schwarz, TJWC.

##### Remarks.

The status of *A.tadornacallida* is unclear and requires investigation; it may well merit species status due to consistent differences in the shape of the genital capsule (see comments in Gusenleitner and Schwarz 2002).

##### Distribution.

*Andrenatadorna* sensu lato is found in Morocco, Algeria, Tunisia, Libya, Egypt, Israel, Jordan, Syria*, Saudi Arabia*, Iraq* Gusenleitner and Schwarz (2002).

#### ﻿52. Andrena (Micrandrena) tkalcui

Taxon classificationAnimaliaHymenopteraAndrenidae

Gusenleitner & Schwarz, 2002 *

6BBACF2D-442B-52E9-9B23-2A84F285D776

##### Material examined.

**Iraq**: Duhok, Bessre [Besereh], Bablo, 36.8675°N, 43.1206°E, 1065 m, 5–6.v.2023, 1♀, leg. D. Baiocchi, MSVI; Duhok, Mt. Gara [S of Sarsing], 37.0158°N, 43.3506°E, 1912 m, 11.v.2023, 1♀, leg. D. Baiocchi, MSVI.

##### Distribution.

Israel and West Bank, Jordan, Syria, Turkey, Iraq*, Iran (Gusenleitner and Schwarz 2002; Wood and Monfared 2022).

#### ﻿53. Andrena (Cordandrena) torda

Taxon classificationAnimaliaHymenopteraAndrenidae

Warncke, 1965 *

B9C9B14C-81B0-5CDA-BA0A-C78FF6808294

##### Material examined.

**Iraq**: Duhok, Mt. Gara [S of Sarsing], 37.0158°N, 43.3506°E, 1912 m, 11.v.2023, 6♂, leg. D. Baiocchi, MSVI/TJWC; **Lebanon**: Beqaa, Beqaa valley, Mansourah, Aammiq wetland preserve, 33.7321°N, 35.7853°E, 850 m, 3.iv.2023, 2♀, leg. T.J. Wood, TJWC; Beqaa, Beqaa valley, Qaraoun, 3.5 km W of Madjal Balhis, 33.5377°N, 35.7038°E, 900 m, 4–5.iv.2023, 2♀, leg. T.J. Wood, TJWC.

##### Remarks.

Records from Lebanon, Jordan, and Syria reported by Wood et al. (2020) were incorrectly identified female specimens of *A.cypria* (see Wood and Monfared 2022), with females of *A.torda* and *A.cypria* being challenging to separate morphologically. The female Lebanese specimens presented here were confirmed as *A.torda* through DNA barcoding (BIN: BOLD:AES5002) as distinct from *A.cypria* (BIN: BOLD:AFH0814), this latter BIN containing a barcoded male specimen of *A.cypria* which can be unambiguously recognised due to the combination of its antennal segment ratios and genital capsule.

##### Distribution.

Greece, Turkey, Cyprus, Israel and West Bank, Lebanon*, Iraq*, Iran (Wood and Monfared 2022).

#### ﻿54. Andrena (Notandrena) trimarginata

Taxon classificationAnimaliaHymenopteraAndrenidae

(Radoszkowski, 1886)

77F8E2C3-A4DB-5F4A-80DE-94AF422DD36F

##### Literature records.

Wood and Monfared (2022, as *A.zostera* Warncke, 1975).

##### Material examined.

**Lebanon**: Balbek-Hermel, Sefri, Haouch Snaid, AUB farm, 33.9244°N, 36.0754°E, 1000 m, 6.iv.2023, 4♂, leg. T.J. Wood, TJWC.

##### Remarks.

A taxonomic work will soon recognise *Halictustrimarginatus* Radoszkowski, 1886 (described from Turkmenistan) as the senior name of *A.zostera* (Wood, in press).

##### Distribution.

Israel and West Bank, Syria, Lebanon*, Turkey, Azerbaijan, Iraq, Iran, Turkmenistan, Uzbekistan, Tajikistan (Osytshnjuk et al. 2005 as *A.subsmaragdina* Osytshnjuk, 1984; Wood and Monfared 2022).

#### ﻿55. Andrena (Aciandrena) turmalina

Taxon classificationAnimaliaHymenopteraAndrenidae

Pisanty & Wood, 2022 *

F90FE70F-B66B-5A3E-A5FA-8DD58DFE6448

##### Material examined.

**Iraq**: Duhok, Bessre [Besereh], Bablo, 36.8675°N, 43.1206°E, 1065 m, 5–6.v.2023, 1♀, leg. D. Baiocchi, MSVI; Duhok, Mt. Gara [S of Sarsing], 37.0158°N, 43.3506°E, 1912 m, 11.v.2023, 4♂, 5♀, leg. D. Baiocchi, MSVI/TJWC; **Lebanon**: Beqaa, Anjar, 1 km E, reforestation area, 33.7311°N, 35.9478°E, 1000 m, 7.iv.2023, 1♀, leg. T.J. Wood, TJWC.

##### Remarks.

As suspected by Pisanty et al. (2022a), *A.turmalina* is also present in Lebanon.

##### Distribution.

Israel, Lebanon*, Turkey, Iraq*, Iran (Pisanty et al. 2022a).

#### ﻿56. Andrena (Holandrena) variabilis

Taxon classificationAnimaliaHymenopteraAndrenidae

Smith, 1853

A6CAE1EF-4242-5FE4-8F17-182B8D48508F

Andrena bakrajoensis Amin & Mawlood, 2019, syn. nov.

##### Literature records.

Amin and Mawlood (2019, as *A.bakrajoensis*)

##### Remarks.

Amin and Mawlood (2019) described *A.bakrajoensis* from Bakrajo in Iraqi Kurdistan. They diagnosed it against A. (Simandrena) vetula Lepeletier, 1841, but based on the description and the provided photographs it is clearly a member of the subgenus Holandrena Pérez, 1890 due to the predominantly declivous propodeum (almost without a dorsal horizontal area), strong tergal hairbands, and compact body with rounded head. Based on the long ocelloccipital distance (3× the diameter of a lateral ocellus), it can only be a single species, *A.variabilis*. *Andrenabakrajoensis* is therefore synonymised with *A.variabilis*. The distribution maps of Gusenleitner and Schwarz (2002) do not positively indicate the presence of *A.variabilis* in Iraq, though some dots are present around the Tigris and Euphrates deltas in southern Iraq. However, *A.variabilis* is a widely distributed species in Turkey and Iran (Gusenleitner and Schwarz 2002; Wood and Monfared 2022), and so its presence in Iraq is expected and demonstrated through this synonymy.

#### ﻿57. Andrena (Planiandrena) veterana

Taxon classificationAnimaliaHymenopteraAndrenidae

Pisanty, 2022 *

4FAE5E3A-2B16-5C56-8298-30E22C0626A8

##### Material examined.

**Iraq**: Duhok, Mt. Gara [S of Sarsing], 37.0158°N, 43.3506°E, 1912 m, 11.v.2023, 1♀, leg. D. Baiocchi, MSVI; **Lebanon**: Beqaa, Rachaiya, 5 km S, Mount Hermon nature reserve, 33.4586°N, 35.8395°E, 1500 m, 8.iv.2023, 10♀, leg. T.J. Wood, TJWC.

##### Remarks.

These records markedly expand the range of *A.veterana* which was described from Mount Hermon (Pisanty et al. 2022a).

##### Distribution.

Israel, Lebanon*, Iraq* (Pisanty et al. 2022a).

#### ﻿58. Andrena (Simandrena) vetula

Taxon classificationAnimaliaHymenopteraAndrenidae

Lepeletier, 1841

8B2E88CA-E4FC-5120-8864-466D307B5FF2

##### Literature records.

Morice (1921b); Derwesh (1965), mentioning “Survey of Iraq Fauna 1915–1919”; Gusenleitner and Schwarz (2002: dot map 504); Augul (2018); Ascher and Pickering (2023).

##### Remarks.

We have not examined any specimens of this species, but the presence of this species in Iraq is highly plausible based on its known global distribution, the map records indicated by Gusenleitner and Schwarz (2002), and the presence of this species in neighbouring Turkey and Iran.

##### Distribution.

West and Central Palearctic (Gusenleitner and Schwarz 2002; Wood and Monfared 2022; Ascher and Pickering 2023).

#### ﻿59. Andrena (Poecilandrena) viridescens

Taxon classificationAnimaliaHymenopteraAndrenidae

Viereck, 1916

10EBF906-AD82-5AC9-B274-DF2EC99429EA

##### Literature records.

Morice (1921b); Derwesh (1965), mentioning “Survey of Iraq Fauna 1915–1919”, as *A.cyanescens* Nylander, 1852 nec. Haliday; Augul (2018).

##### Material examined.

**Iraq**: Duhok, Mt. Gara [S of Sarsing], 37.0158°N, 43.3506°E, 1912 m, 11.v.2023, 5♂, leg. D. Baiocchi, MSVI/TJWC.

##### Remarks.

Members of the subgenus Poecilandrena Hedicke, 1933 are challenging to identify (e.g., Pisanty et al. 2018), but we can confirm the record of Morice (1921b) through the examination of newly collected material.

##### Distribution.

Europe to Turkey, Iraq, Iran (Gusenleitner and Schwarz 2002; Wood and Monfared 2022).

### ﻿Species excluded

#### ﻿ Andrena (Melandrena) sigiella

Taxon classificationAnimaliaHymenopteraAndrenidae

Gusenleitner, 1998

6AD38CDC-C4EA-572F-AC91-55CF316BE693

##### Literature records.

Grace (2010); Augul (2018); Ascher and Pickering (2023).

##### Remarks.

We have examined no specimens of this species from outside of the Levant (Israel and West Bank, Jordan, Lebanon, Syria; Wood et al. 2020). Neither Grace (2010) or Augul (2018) report precise specimen records and, pending further investigations, we exclude *A.sigiella* from the list of Iraqi *Andrena* as if truly present it would be a considerable range extension from the Levant.

#### ﻿ Andrena (Suandrena) leucocyanea

Taxon classificationAnimaliaHymenopteraAndrenidae

Pérez, 1895

FCAB8D87-CE49-5E85-8A82-FB6C6ED4107B

##### Literature records.

Dylewska (1983).

##### Remarks.

Dylewska (1983: 22) revised members of the subgenus Suandrena Warncke, 1968, mentioning *A.leucocyanea* from Iraq from Abu Ghuraib [= Abu Ghraib] and Dewania [= Al Diwaniyah]. Given the many taxonomic changes that have occurred in this subgenus since 1983 (e.g., Kratochwil 2021; Pisanty et al. 2022a) and its difficulty of identification, it is unclear what these specimens might actually be. Consequently, we exclude this species from the Iraqi fauna pending inspection of material in light of the currently accepted taxonomic framework for this subgenus.

## ﻿Discussion

As a measure of its chronic lack of study, the updated faunal list presented here for Iraqi *Andrena* totals some 59 species, a tiny fraction of the 215 and 388 species known from neighbouring Iran and Turkey, respectively (Wood and Monfared 2022; Wood 2023; Wood, unpublished data), and even of the 154–166 species known from neighbouring Syria. Moreover, to the best of our knowledge, the current work represents the first country records for 42 of the 59 Iraqi *Andrena* species reported here. Indeed, the collection around Duhok in May 2023 produced 38 new country records just for *Andrena*, including the two new species for science. Of these 38 new country records, 12 of these species have been described since 2016, highlighting that not only is Iraq understudied in general, but that it specifically hosts a special *Andrena* fauna containing range-restricted species that has received relatively little attention until recently.

The increase from 17 to 59 *Andrena* species represents a 347% increase in species richness. Should it be applied equally across the entire Iraqi bee fauna, the 101 species listed by Ascher and Pickering (2023) would become 350. Though this is a crude approach, this produced total is plausible and seems reasonable given the huge diversity of bee species known to occur in Turkey (1,786 species as listed by Ascher and Pickering 2023). If anything, it is likely to be an underestimate. Based on the initial determination of other Iraqi bee groups, we will soon report faunal increases of 380% for *Eucera* (Apidae), ~ 666–833% for osmiine bees (Megachilidae), and an estimated 430–530% increase for *Nomada* (Apidae) (M. Selis, unpublished data). When dealing with such a poorly studied but likely species-rich fauna, these massive increases indicate a consistent pattern. We hope that this first focused revision of Iraqi *Andrena* can serve as a model to promote further studies into the Iraqi bee fauna.

## Supplementary Material

XML Treatment for Andrena (Aenandrena) aeneiventris

XML Treatment for Andrena (Notandrena) aerinifrons

XML Treatment for Andrena (Taeniandrena) afzeliella

XML Treatment for Andrena (Melandrena) albifacies

XML Treatment for Andrena (Truncandrena) albopicta

XML Treatment for Andrena (Melandrena) albopunctata

XML Treatment for Andrena (incertae sedis) antilibanotica

XML Treatment for Andrena (Chlorandrena) astica

XML Treatment for Andrena (Notandrena) ayna

XML Treatment for Andrena (Notandrena) baiocchii

XML Treatment for Andrena (Plastandrena) bimaculata

XML Treatment for Andrena (Cryptandrena) brumanensis

XML Treatment for Andrena (Truncandrena) caneae

XML Treatment for Andrena (Micrandrena) cedricola

XML Treatment for Andrena (Chlorandrena) cinereophila

XML Treatment for Andrena (Cordandrena) cordialis

XML Treatment for Andrena (Poecilandrena) crassana

XML Treatment for Andrena (Aciandrena) curviocciput

XML Treatment for Andrena

XML Treatment for Andrena (Aciandrena) duhokensis

XML Treatment for Andrena (Micrandrena) elam

XML Treatment for Andrena (Lepidandrena) elisaria

XML Treatment for Andrena (incertae sedis) euzona

XML Treatment for Andrena (Plastandrena) eversmanni

XML Treatment for Andrena (Hoplandrena) ferox

XML Treatment for Andrena (Melandrena) flavipes

XML Treatment for Andrena (Bryandrena) florea

XML Treatment for Andrena (Ulandrena) fulvitarsis

XML Treatment for Andrena (incertae sedis) garrula

XML Treatment for Andrena (Melandrena) grandilabris

XML Treatment for Andrena (Euandrena) hermonella

XML Treatment for Andrena

XML Treatment for Andrena (Poecilandrena) hybrida

XML Treatment for Andrena (Graecandrena) hyemala

XML Treatment for Andrena (Ulandrena) kriechbaumeri

XML Treatment for Andrena (Poecilandrena) laticeps

XML Treatment for Andrena (Melandrena) limata

XML Treatment for Andrena (Micrandrena) luscinia

XML Treatment for Andrena

XML Treatment for Andrena (Melandrena) morio

XML Treatment for Andrena (Melandrena) nitidemula

XML Treatment for Andrena (Micrandrena) obsidiana

XML Treatment for Andrena (Micrandrena) oedicnema

XML Treatment for Andrena (Pallandrena) pallidicincta

XML Treatment for Andrena (Truncandrena) pareklisiae

XML Treatment for Andrena (Truncandrena) rufomaculata

XML Treatment for Andrena (Suandrena) savignyi

XML Treatment for Andrena (Micrandrena) sillatahistrionica

XML Treatment for Andrena (Euandrena) symphyti

XML Treatment for Andrena (Chlorandrena) tadauchii

XML Treatment for Andrena (Ulandrena) tadornacallida

XML Treatment for Andrena (Micrandrena) tkalcui

XML Treatment for Andrena (Cordandrena) torda

XML Treatment for Andrena (Notandrena) trimarginata

XML Treatment for Andrena (Aciandrena) turmalina

XML Treatment for Andrena (Holandrena) variabilis

XML Treatment for Andrena (Planiandrena) veterana

XML Treatment for Andrena (Simandrena) vetula

XML Treatment for Andrena (Poecilandrena) viridescens

XML Treatment for Andrena (Melandrena) sigiella

XML Treatment for Andrena (Suandrena) leucocyanea

## ﻿Additional information

### Author ORCIDs

Thomas J. Wood https://orcid.org/0000-0001-5653-224X

Marco Selis https://orcid.org/0000-0002-8200-9441

### Data availability

All of the data that support the findings of this study are available in the main text.
